# Special Issue of Materials focusing on “Finite Element Analysis and Models of Sustainable Manufacturing Processes”

**DOI:** 10.3390/ma15031116

**Published:** 2022-01-31

**Authors:** Claudio Giardini, Gianluca D’Urso

**Affiliations:** Department of Management, Information and Production Engineering, University of Bergamo, Via Pasubio 7/b, 24044 Dalmine, BG, Italy; gianluca.d-urso@unibg.it

We believe that the chosen topic is nowadays extremely current and of great interest. Indeed, one of the biggest problems of our time is how to produce goods with the lowest possible environmental impact. Therefore, it is important to study new manufacturing processes, or modify the classic ones, or even redesign the products, to reduce the amount of raw material and energy required, thus increasing the efficiency of the process and, at the same time, decreasing the impact on the environment.

The aim of this Special Issue was to collect the research results dealing with the use of finite element analysis in studying new processes or products compliant with sustainable manufacturing requirements.

The six original research papers collected in this thematic issue cover different manufacturing technologies and different parts of production, but are, at the same time, interconnected to meet the ever-growing needs for a safer and less polluted environment.

In particular, in “Thermal Modeling of the Port on a Refining Furnace to Prevent Copper Infiltration and Slag Accretion” [1], the original design of a furnace port was analyzed and modified obtaining a reduction of the average temperature of the critical areas up to 300 K. This is important not only in terms of heating energy required by the furnace, but also in preserving its port plates from being attacked by copper.

In “Simulation of the Mechanical Behaviour of Metal Gyroids for Bone Tissue Application” [2], the background and the steps to build a numerical simulation to extract the mechanical behaviour of the metal gyroids are presented; the research conducted allows the reduction the experimental effort, improving the ability of realizing sound parts (less waste of energy and material).

In “FEM and Analytical Modeling of the Incipient Chip Formation for the Generation of Micro-Features” [3], the target application studied was the sustainable manufacturing of gecko adhesives by means of micromachining an injection molding die. The difficulties of this operation include undercuts and sharp tips. Moreover, in this case, a reduction in environmental impact, in terms of material and energy, can be achieved if the production of sound parts is guaranteed.

In “The Experimental Process Design of Artificial Lightweight Aggregates Using an Orthogonal Array Table and Analysis by Machine Learning” [4], the results of a research aimed to experimentally design the drying, calcination and sintering processes of artificial lightweight aggregates are reported. This is achieved through the orthogonal array, to expand the data using the results, and to model the manufacturing process of lightweight aggregates through machine-learning techniques.

In “Redesign of a Piston for a Diesel Combustion Engine to Use Biodiesel Blends” [5], research on a new-concept piston is presented. This paper shows how (by means of the numerical simulations carried out), even if the base piston and the new one have similar behaviors, with the proposed piston, the effort and the material can be reduced.

Finally, in “Integrated Computational Material Design for PMC Manufacturing with Trapped Rubber” [6], a study about the use of continuous fiber polymer matrix composites expanding into new fields is presented. Additionally, in this case, the requirement is a growing need for more sustainable manufacturing processes. The integrated computational material design framework developed by the Authors will enable the design of tailored manufacturing systems for polymer matrix composite materials in order to achieve high-quality components.

We hope this thematic issue can give a contribution in stimulating further research in the field of energy and material saving, allowing the development of eco-friendly production technologies and components.
